# The Therapeutic Use of Phosphate of Bismuth

**Published:** 1881-03

**Authors:** 


### THE THERAPEUTIC USE OF PHOSPHATE OF BISMUTH.

According to the *Union Pharmaceutique*, Dr. Tédenat prefers the phosphate of bismuth to the sub-nitrate. The anti-diarrhœic action of the phosphate is identical with that of the sub-nitrate. Thanks to its greater insolubility, however, the phosphate acts in slightly less doses, especially in affections of the stomach. In spite of the acidity of the fluids of the stomach, it is completely absorbed.

The dose varies according to the case; generally it is from 1 to 2 grammes. The mode of administration is the same as that of the sub-nitrate. In children it suffices to deposit the desired quantity on the tongue, and give the child the breast or the bottle. The salt is easily drawn into the stomach, and considerable doses may be given in this manner. In adults the remedy is held in suspension in some liquid. In many cases, lozenges of 1 to 2 grammes in weight are very useful; they disintegrate in the mouth, and the phosphate is gradually taken into the stomach.—*Medical Press and Circular.*

